# The Influence of Alcohol on the Opsonic Power of the Blood

**Published:** 1908-02

**Authors:** Chas. E. Stewart

**Affiliations:** Professor Theory and Practice of Medicine, American Medical Missionary College


### Abstracts and Selections.

### The Influence of Alcohol on the Opsonic Power of the Blood.

BY CHAS. E. STEWART, M. D.,
Professor Theory and Practice of Medicine, American Medical Missionary College.

That the *vis medicatrix naturae* is our chief aid in our efforts against disease is recognized by every successful practitioner of medicine, and anything that will help us to secure this condition in its fullest degree will be gladly welcomed and given first place in our therapeutic armamentarium. We are well aware that many diseases are self-limited and if left alone will run their course and finally a normal condition of health obtain; in fact, not infrequently as far as the disease from which the patient suffered is concerned, his health is above par, if such an expression is permissible, for there has been established a condition of immunity which makes the patient much less susceptible to the disease than before he contracted it. Thus we see that the body has within itself certain resources which can be called upon to assist in securing and maintaining a healthy condition.

In the majority of acute diseases these resources are sufficient, and in those cases in which they are not, it is probably due to the fact that the vital forces of the body were at such a low ebb at the time of the onset of the disease, or have become so during its course, that the tissues are incapable of producing the necessary protective substances. That the body has somewhere within its confines the ability under favorable conditions to produce protective substances which render the organism capable of combating diseased conditions has been recognized for a great many years, but it has only been within a comparatively short time that any exact information concerning this interesting fact has been obtained.

In endeavoring to become familiar with these agencies which have to do with the restoration and maintenance of health, the blood naturally suggests itself as being the chief medium through which these agencies act. "The blood is the life," and is the medium in which the life-giving principles of the food we eat and the air we breathe are conveyed to every part of our bodies, and in turn it assist in the removal of the wastes incident to the oxidation which is constantly taking place. In addition to these important functions it also acts as the chief healing and protective tissue of the body.

Since the discovery that bacteria in many instances cause disease, some very important facts have been demonstrated whereby the blood has been shown to play an important rôle as the chief natural defense in protecting the body against disease. It has been clearly demonstrated by Metchnikoff and many others that the leucocytes of the blood play an important part in ridding the organism of bacteria which so frequently gain entrance. The fact that in most infectious disorders there is an increase in the number of leucocytes, and that in many instances the specific bacteria have been found in large numbers in the leucocytes, naturally gave rise to the theory that the leucocytes were the principal agents concerned in protecting the body against disease by their phagocytic action.

Twenty years ago Nuttall and Pflueger demonstrated that the blood contained some substance which was capable of destroying some forms of bacteria.

In 1895 Leclef and Denys were the first to call attention to the influence exerted by blood serum on the phagocytic action of the leucocytes. By their experiments they demonstrated that the blood serum contained some principle which acted upon the bacteria in such a way as to render them more susceptible of being acted upon by the leucocytes, thereby materially increasing phagocytosis.

In 1897 Mennes demonstrated that the immunity conferred upon certain animals when inoculated with immunizing sera or bacteria depended upon a modification of their serum in such a manner that as a result a more liberal phagocytosis was brought about without any special activity on the part of the leucocytes. That there are several substances in the blood which influence bacteria in such a way as to destroy them or render them capable of destruction by the leucocytes has been amply demonstrated. The name agglutinin has been given to the substance formed in the blood as a result of bacterial invasion and capable of causing an agglutination of the bacteria producing the infection. The term paralysis has also been used to designate the same principle. Such a principle is found in the blood in cases of typhoid fever, and is the basis for the Widal test which is rendering such valuable service in the diagnosis of this disease. Substances which combine with the bacteria and accomplish their destruction, and designated as bacteriolysin, are found in the blood as a result of bacterial invasion. Still other substances are found which seem to have a special affinity for the bacterias, combining chemically with them so that they are much more readily taken up by the leucocytes. To these substances the term bacteriotropic has been applied.

In 1903 Wright and Douglas, of London, England, used the word opsonin (from the Latin *opsono:* "I cook for the table," "I prepare pabulum for") to indicate certain bacteriotropic substances formed in the blood which act upon bacteria in such a way as to render them subject to injestion by the leucocytes. They have also worked out a practical laboratory technic by means of which the opsonins can be measured with a considerable degree of accuracy, thereby making it possible to approximately estimate one's ability to resist bacterial invasion.

In order to determine the opsonic power of a patient's serum as compared with a normal individual (the opsonic index), Wright and Douglas have made this comparatively easy. Without entering into the details of the technic it may be briefly described as follows: equal volumes of an emulsion of bacteria to be used in salt solution; washed white corpuscles, and the serum to be tested are measured in a long capillary pipette, these are thoroughly mixed, then drawn into the pipette, the end sealed in a flame, and then placed in an incubator for fifteen minutes at 37 degrees C. The pipette is then removed from the incubator, the seal broken and a drop of the mixture placed on a clean slide, and a smear made which is then fixed and stained. The slide is then placed under an oil immersion lens and the number of bacteria found in from 100 to 200 polymorphonuclear neutrophiles are estimated, and from this the average per leucocyte is obtained. Another estimation is made in the same manner except that the serum from a normal individual is used. The ratio of the first average to that for the normal serum constitutes the "opsonic index."

Since the practicability of obtaining the "opsonic index" with a considerable degree of accuracy has been so clearly and cleverly demonstrated, numerous investigators have been busily engaged in trying to prove or disprove Wright's claim that "we have, in the power of raising the antibacterial power of the blood with respect to any invading microbe, out of all comparison the most valuable asset in medicine." Since the opsonins of the blood play such an important part in assisting the leucocytes to rid the body of bacteria, it will be readily inferred that any substance which, when introduced into the body, is capable of modifying this index, will either increase or decrease its vital resistance and, in so doing, will be a factor in combating or inviting disease.

To those substances which when introduced into the organism increase the elaboration of protective substances the term vaccine has been given. In many instances these vaccines are derived from bacterial protoplasm.

Wright and Douglas have discovered that by the administration of bacterial vaccines, in many instances the patient's opsonic index can be raised, and as long as the index can be kept at a normal point or above, the prospects for ultimate recovery are good. However, the converse is true that any substance, whether bacterial vaccine or drug, that is introduced into the organism, lowers the opsonic index and by so doing lowers vital resistance, thereby inviting bacterial invasion, lessens the chances for recovery. Professor Wright further states that "there exists in normal blood, and there exists in a larger quantity in the serum of the successfully inoculated patient, an element which enters into chemical combination with the staphylococcus, the tubercle bacillus, or other micro-organisms in such a manner as to prepare it for phagocytosis. We have demonstrated that phagocytosis can not take place, apart from the action exerted by the specific opsonin upon the micro-organism." Just how these opsonins are produced is not known, but when we find a patient's specific opsonic power persistently low, we are justified in concluding that he is not improving, and we realize that more opsonic power is needed in order to render the invading germs favorable for ingestion by the leucocytes. In most cases of bacterial invasion an increase in the number of leucocytes is greatly to be desired, but an increase in the opsonic power of the blood is still more important; for, as Professor Wright has said, "we have demonstrated that phagocytosis can not take place, apart from the action exerted by the specific opsonin upon the micro-organism."

Therefore a patient suffering from some acute infectious disorder, such as lobar pneumonia, in whose blood there is a good degree of leucocytosis and whose specific opsonic power remains normal or above, can safely be considered as making satisfactory progress toward recovery, and any measure which will encourage and maintain such conditions should be considered eminently satisfactory; and conversely, any measure which will more or less permanently lower the specific opsonic power of the blood, must be considered as inimical to the welfare of the patient and should be abandoned at once.

Without entering into details, we have in these few paragraphs endeavored to outline the fundamental principles pertaining to the determination of the opsonic index and the practical value of this knowledge as applied in the treatment and prevention of disease.

A few weeks ago it was the good fortune of the writer to meet Professors Wright and Douglas and to become somewhat familiar with their work in St. Mary's Hospital, and we must say that if the results to be obtained from the practical application of this theory are commensurate with the enthusiasm over the results already obtained, especially by Wright and Douglas, there has dawned a new era in the treatment of disease and in prophylaxis which is unsurpassed by any previous line of therapeutics, and we are in full accord with the statement of Professor Wright already quoted to the effect that "we have, in the power of raising the antibacterial power of the blood with respect to any invading microbe, out of all comparison the most valuable asset in medicine."

During a lecture by Professor Wright, in considering the agencies which modify phagocytosis, among others he mentioned alcohol, and stated that the leucocytes were "antitemperance," because, when freed from blood serum and then soaked in alcohol, phagocytosis is increased. This statement greatly surprised me, for from practical experience with diseases such as tuberculosis and pneumonia, where phagocytosis plays such an important part in ridding the body of bacteria, I had discovered that when alcohol was withdrawn and measures instituted to encourage leucocytosis, and improve the general vital resistance, such as the cold air treatment, tonic hydriatic measures and plenty of sunshine, marked improvement was noted. I recalled the statement of Professor Wright, that "phagocytosis can not take place apart from the action exerted by the specific opsonin upon the micro-organism," and concluded that in all probability the evil effects of alcohol were due to its lowering the opsonic power of the blood.

In order to determine the facts with reference to this the medical literature was carefully searched, but nothing was found pertaining to the influence of alcohol upon the opsonic power of the blood in the living organism. A series of experiments were at once instituted for the purpose of ascertaining, if possible, what influence the administration of alcohol has upon the opsonic power of the blood in healthy individuals, and while in the short time which has elapsed since they were started, only a comparatively few have been performed, results obtained so far are quite uniform, and we believe justify us in making this preliminary report, with the hope that it may be a means of encouraging others to pursue the work further in this direction, and if possible help to demonstrate the truth with reference to the influence of alcohol upon the *vis medicatrix naturae.*

Rubin ("The Influence of Alcohol and Chloroform on Phagocytosis in Vitro," *Journal A. M. A.*, Vol. XLVIII, No. 17, page 1432, April 27, 1907), in his study of the effect of alcohol in vitro, employed the following technic: "Suspensions of various bacteria, especially staphylococci, pneumonocci and streptococci are added to defibrinated dog blood containing alcohol or chloroform in varying proportion, the tubes are then carefully corked and placed in an incubator at 37 degrees C. for 30 to 40 minutes. Then smears are made and the bacteria in the leucocytes counted and averaged. In every case control experiments were made with mixtures of normal blood and bacteria. The accompanying table illustrates the results obtained when alcohol was added in varying proportions.

| | Degree of dilution with alcohol | Bacterum | Control | Alcohol Mix. |
|---|---|---|---|---|
| No. 1 | 1 to 1,000 | Staphylococcus | 4.5 | 5.5 |
| No. 2 | 1 to 500 | " | 25 | 14 |
| No. 3 | 1 to 400 | Streptococcus | 4 | 2 |
| No. 4 | 1 to 300 | Pneumococcus | 11 | 3 |
| No. 5 | 1 to 200 | " | 7 | 1 |
| No. 6 | 1 to 100 | " | 26 | 4 |
| No. 7 | 1 to 50 | " | 5 | 0 |

From these experiments it will be seen that one part of alcohol to fifty of serum suspends phagocytosis in vitro completely, and even in such dilutions as one part of alcohol to 500 of serum phagocytic action is reduced 44 per cent.

Our own investigations have been confined to the action of small amounts of alcohol administered internally to persons whose norman opsonic power was .75 or above and who were previously total abstainers, and to the influence of alcohol on phagocytosis in vitro. (Tables omitted.)

For these investigations, in order to secure a standard index, the average from a pool of nine was obtained, and in no instance was blood used in which the index was below 1.00. As a result the average secured is high, being 2.12, consequently the indices of the blood used are correspondingly low. In each case the bacillus tuberculosis was employed.

An examination of this table shows a marked drop in the opsonic power after the addition of even so small an amount of alcohol as 5 per cent, and a correspondingly greater drop when the amount was increased to 10 and 20 per cent.

The average normal index in the normal cases, including that of the pool of nine, is .922; after the addition of 5 per cent of alcohol the average index dropped to .436, showing a loss to the extent of 52.71 per cent; the average index when 10 per cent of alcohol was used was found to be .310, or a loss of 66.37 per cent; when 20 per cent of alcohol was added the average index was .128, showing a loss of 86.11 per cent in the opsonic power.

In summarizing the results of these experiments, we find that in the four cases where alcohol was taken internally in the form of port wine, the opsonic power of the blood was greatly lowered, as is evidenced by a comparison of the normal indices obtained with the bacillus tuberculosis and streptococci as compared with the indices obtained after the ingestion of the wine. The average index for the bacillus tuberculosis in the four cases cited is 1.17, for streptococci 1.12. The average of these same cases after the administration of the two ounces of port wine is .73 and .655, respectively, showing a drop in the opsonic power of 37 per cent in the former and 42 per cent in the latter.

In the three cases in which Peruna was used the average normal index for bacillus tuberculosis was 1.12, for streptococci 1.09; four hours after the ingestion of two ounces of Peruna, the average index in the former was .133, in the latter .68, showing in the former a drop of over 88 per cent in the opsonic power, and in the latter a drop of 36 per cent.

We realize that there are a great many factors which influence the opsonic power of the blood and that of necessity there must be a considerable variation in even what might be considered normal cases, but, notwithstanding these variations, there is a sufficient uniformity to enable us to make some very valuable deductions. From our investigations, as will be seen from a study of the tables, there was in almost every case after the administration of a comparatively small quantity of alcohol, both internally and in vitro, a very marked reduction in the opsonic power. So we feel justified in concluding that alcohol has a marked influence in reducing the vital forces of the body, thereby greatly interfering with the *vis medicatrix naturae*. Since, according to Wright, "out of all comparison the most valuable asset in medicine lies in raising the antibacterial power of the blood," the administration of alcohol, which, according to our investigation is probacterial, and as such is a strong liability instead of an asset, should consequently be eliminated from our therapeutic armamentarium, at least as far as internal administration is concerned in infectious diseases.

In this connection, I wish to extend to Drs. A. W. Nelson and L. Stoner, of the Battle Creek Sanitarium Laboratory, my thanks for their efficient services in conducting the laboratory technic in connection with these investigations.—*Journal of Inebriety.*

